# Association of trinucleotide repeat polymorphisms CAG and GGC in exon 1 of the androgen receptor gene with male infertility: a cross-sectional study

**DOI:** 10.55730/1300-0144.5525

**Published:** 2020-09-10

**Authors:** Mussarat ASHRAF, Shahana Urooj KAZMI, Hemaila TARIQ, Adnan MUNIR, Rehana REHMAN

**Affiliations:** 1 Department of Biological and Biomedical Sciences, Medical College, Aga Khan University, Karachi, Pakistan; 2 Department of Clinical Microbiology and Immunology, Faculty of Science, Dadabhoy Institute of Higher Education, Karachi, Pakistan; 3 Department of Urology, Medical College, Liaquat National Hospital, Karachi, Pakistan

**Keywords:** Androgen receptor, trinucleotide repeats, male infertility

## Abstract

**Background/aim:**

Infertility is a global problem that brings about serious sexual and social consequences that strain the health sector and society. The expansion of CAG and GGC repeats in androgen receptor (*AR*) gene (Ensembl number ENSG00000169083) may lead to reduced fertility. Our objective was to determine the association of CAG and GGC repeats with altered sperm parameters in male infertile subjects.

**Materials and methods:**

This was a cross-sectional study conducted at Aga Khan University, Karachi, Pakistan. A total of 376 males were recruited, out of which group A (N = 208) and group B (N = 168) were comprised of subjects with normal and altered sperm parameters, respectively, from 18 to 60 years. The numbers of CAG and GGC repeats were determined by using PCR amplification and sequence analysis using the Molecular Evolutionary Genetic Analysis (MEGA) software version 6.0. Statistical analysis was performed using the SPSS version 20 and the P-value of <0.05 was considered significant.

**Results:**

The mean androgen receptor gene CAG repeats were significantly longer in males with altered sperm parameters as compared to male subjects with normal sperm parameters (P < 0.001). There was no significant difference found for GGC repeats for subjects with altered sperm parameters.

**Conclusion:**

Longer CAG length corresponded to greater severity of spermatogenic defect and may lead to subfertility recommendations.

## 1. Introduction

The WHO defines infertility as “a disease of the reproductive system defined by the failure to achieve a clinical pregnancy after 12 months or more of regular unprotected sexual intercourse” [1]. Around the world, research on infertility has focused on female infertility, but the recent shift in healthcare dynamics have necessitated a fresh look into male infertility and the corresponding factors that influence its extent, severity, and prevalence. Male infertility in 50% of the cases is idiopathic due to the fact that human spermatogenesis is controlled by several hundreds of genes [2].

The environmental causes of male infertility include prolonged exposure to heat [3], noise pollution [4], ionizing radiation [5], air pollution [6], mumps after puberty [7], alcoholism [8], and psychological stress [9].

The genetic causes underlying male infertility include Y chromosome microdeletions [10], sex chromosome aneuploidies such as Klinefelter’s Syndrome [11], and gene polymorphisms such as those in LOC203413 or CAG repeats in androgen receptor gene [12].

Androgen is indispensable for the male sex differentiation and spermatogenesis [13]. Testosterone and dihydrotestosterone are physiological androgens that play an important role in the development of male external and internal genitalia [14]. Androgens act through a steroid receptor known as androgen receptor, which is encoded by an androgen receptor gene, a ligand dependent nuclear transcription factor [15]. The androgen receptor (*AR*) gene (Ensembl number ENSG00000169083) is located on chromosome Xq11-12 and has 8 exons and 7 introns, out of which exon 1 has CAG and GGC repeats and is associated with transcription [14,16].

Polymorphic trinucleotide repeat segment (CAG)n encodes a polyglutamine tract in which n usually ranges from 11 to 31. This CAG repeat tract has been the basis of unprecedented attention in current years because it has been found that the expansion of the CAG segment to more than 40 repeats leads to spinal bulbar muscular atrophy, a mortal neuromuscular disease. Trinucleotide repeat expansions are now associated with many diseases [14].

The *AR* gene is susceptible to one of the largest number of mutations in a steroid receptor, documented at up till 300 mutations [17]. The *AR* gene essentially consists of three main functional domains as the N-terminal transcriptional regulation domain, the DNA-binding domain, and the ligand-binding domain. Androgens bind to the *AR* gene and exert their actions either through DNA binding-dependent manner, regulating gene transcription, or non-DNA binding-dependent manner, causing phosphorylation of the secondary messenger system [18]. The *AR* gene is expressed on a multitude of tissues in the body such as prostate, bone, adipose tissue, muscle, etc., and plays an important role in the immune, hematopoietic, respiratory, cardiovascular, and neural systems [19,20].

The CAG trinucleotide repeat length is normally between 16 and 29 [21]. The variation in the length of CAG repeats is between 6 and 39, with African Americans having an average length of 19 to 20 repeats, Caucasians having 21 to 22, Asians having 22 to 23, and Hispanics having 23 repeats [22]. CAG, located within the N-terminal transactivation domain, encodes polyglutamine and the number of glutamine encoded is significant for the function and structure of the *AR* gene [23] since the number of the CAG triplet repeats determines the variability of the size of the androgen receptor [24].

*AR* resistance is reported to account for 40% of male infertility cases [25]. Increased CAG length in the *AR* gene has been correlated with male infertility owing to a decreased transcription of the *AR* gene and diminished spermatogenesis [26]. Therefore, an inverse relationship exists between the longer CAG repeat length and the transcriptional activity of the *AR* gene [27]. Most of the infertile males with an increased CAG length presented with azoospermia [13]. Even though studies show that shorter CAG repeat lengths such as in African Americans predispose them to developing prostate cancer, it has been reported that increased CAG repeat lengths have been attributed to male infertility cases among Asians and Caucasians [22,28,29]. An increased CAG length of >26 nucleotides is linked to a 7-fold increased risk of infertility [30]. An increased CAG repeat length is also associated with depression and reduced potency in males and a CAG repeat length greater than 40 is known to be linked to Kennedy’s disease, a fatal neuromuscular disease [31,32]. However, the exact molecular mechanism behind CAG repeat polymorphism along with the association between the length of CAG repeats and the severity of disease requires further studies [33].

The transactivation domain of AR also contains a polyglycine tract, encoded by GGT/GGC six-glycine tract proceeded by a polymorphic GGC repeat of a length of 10 to 35 nucleotides. In spite of the limited studies showing the association between GGC repeat polymorphism and male fertility, there have been certain studies [34] showing an association between GGC and the *AR* gene protein levels, suggesting that the GGC repeat length of 13 produced 2.7 times more AR proteins than the GGC repeat length of 17 did. A shorter GGC repeat length has also been linked to prostate cancer [35]. Research shows that both long CAG and GGC repeats have a negative impact on transcriptional activity of the *AR* gene, while also having an association with the prostate and endometrial cancers [36]. Hence, we are interested in finding the association between the lengths of the CAG and GGC repeats in the *AR* gene and altered sperm parameters in our population of male subjects with infertility problems.

## 2. Materials and methods

This cross-sectional study was conducted from July 2017 to July 2018 at the Department of Biological and Biomedical Science, Aga Khan University, in collaboration with the Sindh Institute of Reproductive Medicine after acquiring the ethical approval of the institutional ethical review board (2019-1226-3970). The sample size was calculated on the Open Epi software version 3.01. A sample size of 398 was estimated to find the CAG and GGC repeat polymorphism in both groups adjusted for 10% nonresponse rate. We assumed a level of significance of 5%, an odds ratio of 2, and power for detecting the true effect of 80%. However, the final recruitment was 376 males on account of a number of refusals to provide semen samples.

### 2.1. Patient and public involvement

All the male subjects were recruited from the Sindh Institute of Reproductive Medicine based on nonconception after regular, unprotected intercourse for a period of at least 1 year. Possible genetic causes of male infertility were excluded by Y chromosome microdeletions and Karyotype analysis. The infertile subjects were divided into two groups (A and B) on the basis of normal or altered semen parameters, respectively. All the subjects were collected via convenience sampling after obtaining written and informed consent.

At the time of recruitment, a data collection form was filled to record clinical data such as age, height, weight, the calculation of body mass index (BMI), the estimation of body fat percentage, blood pressure, smoking habits, hormonal treatment, and clinical history. Group A (normal sperm parameters) comprised of male subjects with a sperm count more than 39 × 10^6^, sperm motility of more than 50%, and normal morphology ^3^4% with age ranging from 18 to 60 years from all ethnic backgrounds [37]. The male subjects who were smokers, had diabetes, hypertension, or any serious health condition like myocardial infarction or were under hormonal treatment were excluded from the study. The male subjects who had a sperm count less than 39 × 10^6^, a sperm motility less than 50%, and a normal morphology £4% [37] between the ages of 18 and 60 years from all ethnic backgrounds comprised Group B. The exclusion criteria were kept as the same for infertile subjects.

Blood samples were collected for DNA extraction from antecubital vein in 2 tubes, serum gel tube (3 mL) and EDTA tube (2 mL). Serum was centrifuged at 3500 rpm for 5 min, separated into two aliquots, and stored at −70 °C. DNA was extracted from 2 mL of blood in EDTA tube using the standard protocol of Promega Genomic DNA Extraction Kit Cat# A1125. The steps included taking 6 mL of cell lysis solution with 2 mL of blood (centrifuged at 3500 rpm for 10 min), adding 2 mL of nuclei lysis solution and 660 μL of protein precipitation solution (centrifuged at 3500 rpm for 10 min), transferring the supernatant to a tube of 2 mL cold isopropanol, aspirating and adding 200 μL of rehydration solution, incubating for 24 h at 4 °C, vortex before aliquoting the extracted DNA, and keeping it at −80 °C for further analysis. The extracted DNA was quantified by measuring the ultraviolet absorbance and determining the absorbance ratio (A280/A260) for 2-μL samples using a Nanodrop-ND1000 (Thermo Fisher Scientific, Waltham, MA). The extracted DNA was considered pure at an absorbance ratio of ~1.8.

The primers were designed using Primer 3 output primer designing tool for CAG (forward: 5’-TCCAGAATCTGTTCCAGAGCGTGC-3’, reverse: 5’-GCTGTGAAGGTTGCTGTTCCTC-3”) and GGC (Forward: 5’-ACAGCCGAAGAAGGCCAGTTGTAT-3’, reverse: 5’-CAGGTGCGGTGAAGTCGCTTTCCT-3’) repeat region of the *AR* gene. Polymerase chain reaction (PCR) for CAG repeats was performed using 2× PCR HotStart Master Mix (Cat# G906, ABM (Applied Biological Materials Inc, Canada)) as per the manufacturer’s instructions. The PCR conditions were 1 cycle for 5 min at 95 °C for the initial denaturation followed by 30 cycles at 95 °C for 30 s, 60 °C for 45 s, 72 °C for 45 s followed by a final extension of 10 min at 72 °C. PCR was performed for GGC repeats using GoTaq HotStart Master Mix (Cat# M5122, Promega, USA) as per the manufacturer’s instructions. The PCR conditions were 1 cycle for 5 min at 95 °C for the initial denaturation followed by 30 cycles at 95 °C for 30 s, 50 °C for 45 s, 72 °C for 45 s followed by a final extension of 10 min at 72 °C. The amplified products were electrophoresed on 2% agarose gel. The purification of the PCR products was performed using PCR Clean Up for DNA Sequencing (Cat. No BT5100, Bio Basic Inc, Canada) following the manufacturer’s protocol.

Sanger sequencing is a classical method for sequencing and CAG polymorphism in the *AR* gene can be detected by PCR amplification and direct sequencing in the published article [14]. This method was utilized to sequence the *AR* gene in the samples of Group A and B and the PCR products were sent to the sequencing company Operon (Canada). The samples were sequenced to obtain accuracy in finding the number of repeats in polymorphic trinucleotide repeat segment of the *AR* gene. The obtained sequences were directly compared to the previously published CAG and GGC repeat region of the *AR* gene sequence using the MEGABLAST search tool in the National Center for Biotechnology Information (NCBI) database. The sequence files were imported into Chromas Lite, and then assembled using Molecular Evolutionary Genetic Analysis (MEGA) version 6.0.

Statistical analysis was performed with the SPSS version 20 software using the Mann–Whitney U test, and Spearman’s rank correlation tests (P-value of <0.05 was considered significant). Logistic regression analysis with 95% confidence intervals (CIs) was performed to report odds ratios (OR). The sequences were analyzed using Molecular Evolutionary Genetics Analysis (MEGA) software version 6.0.

## 3. Results

A total of 376 males presenting to infertility centers were enrolled in the study. The subjects in Group A (168) had normal semen parameters, whereas 208 male subjects in Group B had abnormal semen parameters.

Table 1 presents the demographic characteristics of both groups. The mean age among Group B was significantly higher (37.4 ± 7.1) as compared to Group A subjects (35.5 ± 6.4, P < 0.001). The mean BMI was significantly higher among the altered sperm parameters (Group B) as compared to the subjects with normal sperm parameters (Group A) (27.6 ± 2.6 vs 24.6 ± 3.2, P < 0.001). The mean body fat % was also significantly higher in Group B as compared to Group A (34.5 ± 4.7 vs 32.9 ± 4.1, P < 0.001).

Table 2 presents the semen characteristic of the subjects in both groups. The mean total count and morphological normal sperms was higher among Group A as compared to Group B, (97.2 ± 30.2 vs 33.4.98 ± 22.6 (P < 0.001) and 8.2 ± 4.1 vs 3.5 ± 2.3 (P < 0.001), respectively). The motility was lower among Group B as compared to Group A subjects with a median (IQR) of 41 (25–46) and 75 (70–78), respectively (P < 0.001). A higher proportion (41.3%) of Group B had Teratozoospermia followed by astheno-Teratozoospermia (16%), azoospermia (16%), severe oligo-astheno-teratozoospermia (SOAT) (15.4%), and lastly oligo-astheno-teratozoospermia (OAT) (10.6%).

Table 3 shows the logistic regression analysis for Group B, which includes males with altered sperm parameters after adjusted with age and BMI. Males who have a shorter CAG length (<26) are considered baseline to find out the odds ratios for sperm count, motility, and morphology for males having a longer CAG (> or = 26). With every one unit of increase in the sperm count, the prevalence of infertility was decreased by 5% (P < 0.001); however, with every one unit of increase in the sperm motility and morphology, the prevalence of infertility was decreased by 18% and 4%, respectively (P < 0.001). Considering the altered sperm parameters as the factor contributing to subfertility (Table 4), the results of the association between the CAG length and male infertile subjects of Group B having longer (> or = 26) and shorter (< 26) CAG lengths are outlined. The sperm count with a CAG length (<26) was 36 million/mL, which was significantly higher than the sperm count with longer CAG lengths (> or = 26), which was 30 million/mL (P = 0.001). Among those with shorter CAG lengths (<26), the sperm motility was 35%, which was significantly higher than the sperm motility among those with longer CAG lengths (> or = 26), which was 21% (P = 0.002). Among those with shorter CAG lengths (<26), the sperm morphology was 3%, which was comparable with longer CAG lengths (> or = 26), which was 2%.

**Figure 1:**
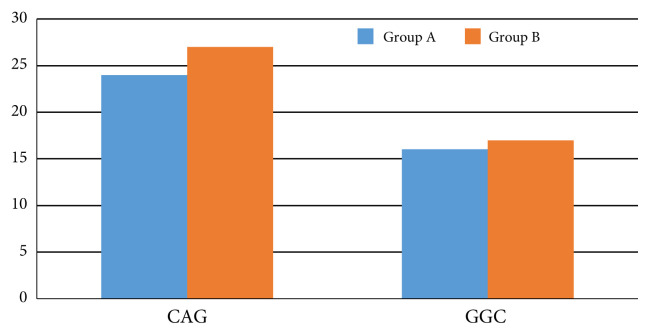
Comparison of mean values of CAG and GGC repeats in normal and altered sperm parameters. * shows that there was significant difference (P < 0.05) between Group A (normal sperm parameters) and Group B (altered sperm parameters) for CAG repeat polymorphisms, using the Mann–Whitney test.

The mean values of the CAG lengths in infertile men with altered sperm parameters (27) were significantly higher than the ones who had normal sperm parameters (24) as shown in Figure 1. There was, however, no significant difference observed between GGC repeat polymorphism in male subjects with normal (A) and altered sperm parameters (B).

**Figure 2:**
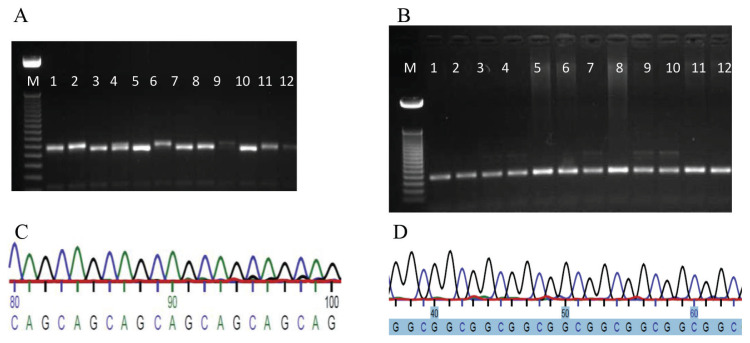
Gel electrophoresis and sequence chromatograms of CAG and GGC repeats in male subjects (Group B). (A–D) Gel electrophoresis and sequencing chromatograms of CAG and GGC repeat polymorphism, where M is the 100bp DNA marker. (A,B) PCR amplification shown as bands on 2% agarose gel of CAG repeats (~288 bp) and GGC repeats (~184 bp) respectively of male samples of Group B numbered 1–12. (C,D): Sequencing chromatograms showing the CAG and GGC repeats, respectively.

Figures 2A and 2B present the gel electrophoresis images of the amplified PCR products for CAG repeats (band size = ~288bp) and for GGC (band size = ~184bp) in infertile and fertile males. Figures 2C and 2D present the sequencing chromatograms of the CAG and GGC repeat polymorphism regions of the *AR* gene.

However, GGC length alone did not show any significant association with male infertility as demonstrated by our results. The mean values of GGC length in the Group B subjects (17) were comparable with the Group A (16) subjects. Figure 2B shows that the most frequently reported GGC length among both the Group A and B subjects was 17, which showed no difference between the two groups.

## 4. Discussion

The increased prevalence of infertility with psychological and economic burdens of developing countries has motivated researchers to look for the relationship of infertility with genetic factors. The main observation in this study was that infertile males have significantly longer CAG lengths compared with fertile male subjects.

Increased fat in the scrotum leads to the production of gonadal heat, which impairs sperm production since sperms need an optimum temperature range of 34–35 degrees. This accounts for the higher BMI among infertile men compared to fertile men in our study. Heat production causes oxidative stress to sperm, the fragmentation of sperm DNA, and diminished sperm motility [38].

An increased CAG trinucleotide repeat length has been associated with male infertility [39]. This is in contrast to few studies such as Nenonen et al., which do not demonstrate a significant association between CAG repeat lengths and male infertility [40]. The CAG trinucleotide length influences the functioning of the androgen receptor gene [41]. CAG tri-nucleotides are located within the N-terminal transactivation domain and encode polyglutamine and the number of glutamine determines the function and structure of the *AR* gene [23]. Increased CAG tri-nucleotide lengths are associated with diminished action of androgen receptor whereas decreased lengths of the CAG trinucleotide are associated with an enhanced action of androgen receptor [42]. Shorter CAG lengths, on the other hand, are demonstrated by studies such as Gomez et al.’s to be associated with the early onset and sporadic prostate cancer. Longer CAG lengths lead to decreased transcriptional activity of the androgen receptor gene and subsequently impair spermatogenesis [24]. Thus, our results show a higher mean value of the CAG length among infertile men compared to fertile men.

The total sperm count and sperm motility of the infertile men recruited in our study was reported to be lower than that of the fertile men recruited, which corroborates the negative effect of the increased CAG length (>26) on sperm parameters. Furthermore, there are studies such as Mengual et al., who showed that men with greater than 26 CAG repeats had a risk of being azospermic [43]. Xiao et al., on the other hand, showed the association between longer CAG lengths and oligospermia, not azoospermia or severe oligospermia. Delli Muti et al. demonstrated that an increased CAG length causes decreased sperm motility, which was proven by our results [44]. Pan et al. showed an inverse relationship between CAG lengths and male infertility among Asian, Caucasian, and mixed races, in conjunction to demonstrating the increased risk of azoospermia with longer CAG lengths [21]. Even though the difference between the sperm morphology among the infertile men and fertile men in this study was not statistically significant, there are studies such as Milatiner et al.’s, which demonstrate a positive correlation between abnormal sperm morphology and increased CAG lengths [45].

However, GGC length alone did not show any significant association with male infertility as demonstrated by our results. Even though studies such as Gao et al.’s show the deletion of polyglycine tract and the reduction of androgen receptor transcriptional activity, there is little evidence to prove the impact of GGC length and male infertility [46,47]. Studies such as Ferlin et al.’s show the combined effect of CAG and GGC in terms of causing male infertility. Furthermore, CAG/GGC haplotypes have demonstrated an association with male infertility, as reported by Ferlin et al. [13]. The aforementioned article also shows that CAG repeats and GGC repeats individually were not significantly different among those with azoospermia, mild or severe oligospermia. However, the haplotype CAG/GGC was reported more frequently among those with a low sperm count. Therefore, there is a need to explore the association between GGC lengths alone and their impact on male infertility. Moreover, the CAG repeats in the *AR* gene is a polymorphism that may be associated (not a causal effect) with male infertility, and its clinical relevance is still debated. The limitations of the present study are that the occupational status of each subject was not determined and we did not correlate smoking status with polymorphism. All triplet repeat disorders show anticipation and a significant correlation between age at the onset of the disease and the length of the expanded repeat [48,49]. The current data is insufficient to conclude whether IVF patients who display *AR* CAG expansion may transfer infertility to their descendants. This CAG triplet repeat disorder (Group B), however, is anticipated to be expressed in future generations in terms of an alteration in sperm count, motility, and morphology, hence contributing to male infertility. *AR* CAG polymorphism is not recommended in the routine setting, yet the test may become imperative on the basis of clinical relevance, pharmacogenetic implications, theoretical possibility of transmission to next generation, and tailoring of testosterone replacement therapy on the basis of length of CAG repeat in hypogonadal men.

Longer AR CAG repeat lengths cause decreased sperm motility, corresponding to a greater severity of spermatogenic defect that can lead to disturbance in reproductive functions, thus causing male infertility. However, GGC did not reflect any association with male infertility.

## Figures and Tables

**Table 1:** Demographic characteristics of male subjects stratified on the basis of sperm parameters.

Variables	Group A normal sperm parameters (n=168)	Group B altered sperm parameters (n=208)	P-value

Age (years) (Mean ± SD)	35.5 ± 6.4	37.4 ± 7.1	<0.001

BMI (in kg/m^2^)		27.6 ± 2.6	
(Mean ± SD)	24.6 ± 3.2	42 (20.2%)	<0.001
BMI (in)	73(43.5%)	166(79.8%)	<0.001
18.5–24.99 kg/m^2^	95(56.6%)		
25 -29.99 kg/m^2^			

Body fat %			
(Mean ± SD )	32.9 ± 4.1	34.5 ± 4.7	
Body fat %	4 (2.4%)	3 (1.4%)	<0.001*
<25	36(21.4%)	29 (13.9%)	0.32
25–29.99	128(76.2%)	176(84.6%)	
>29.99			

Values are expressed as mean ± standard deviation. Groups A and B were compared using the Mann–Whitney test.

* P < 0.05 was considered significantly different.

**Table 2:** Semen characteristics of the study groups.

Factors	Group A normal sperm parameters (n=168)	Group B altered sperm parameters (n=208)	P-value

Total count (in million/mL )	97.2 ± 30.2	33.4 ± 22.6	<0.001*
Mean ± SD			

Motility	75 (70–78)	41 (25–46)	<0.001*#
Median (IQR)			

Morphology	8.2 ± 4.1	3.5 ± 2.3	<0.001*
Mean ± SD			

Morphological forms			<0.001*^
Normozoospermia	135 (100.0%)	0 (0%)	
Teratozoospermia	0 (0%)	86 (41.3%)	
Azoospermia	0 (0%)	34 (16.3%)	
Severe oligo-astheno-teratozoospermia (SOAT)	0 (0%)	32 (15.4%)	
Astheno-teratozoospermia	0 (0%)	34 (16.3%)	
Oligo-astheno-teratozoospermia (OAT)	0 (0%)	22 (10.6%)	

Rapid linear Progression	1.00 (0–2)	0.00 (0–1)	0.015*#
Median (IQR)			

* Significant at P < 0.05 (independent t test was used for quantitative variables).

*^ Significant at P < 0.05 (Fisher’s exact test was used).

*# Significant at P < 0.05 (the Mann–Whitney test was used).

**Table 3:** Effect of sperm characteristics on chances of subfertility.

Factors	Odds ratio	95% confidence interval	P-value
Total sperm count (million/mL)	0.995	0.992–0.999	<0.001
Sperm motility (%)	0.982	0.977–0.997	<0.001
Sperm morphology (%)	0.960	0.952–1. 024	<0.001

* Significant at P < 0.05 (Logistic regression analysis was performed to find odds ratio and 95% confidence interval for risk of male subfertility).

**Table 4:** Association of sperm parameters with CAG length in infertile males with altered sperm parameters (n=208).

Factors	CAG (<26) n=96	CAG (≥26) n=112	rho value	P-value
Total sperm count (million/mL)	36.4 ± 24.4	30.1 ± 21.4	− 0.444	0.001*
Sperm motility (%)	35.4 ± 21.04	21.3 ± 12.1	−0.326	0.002*
Sperm morphology (%)	3 ± 1.6	2 ± 0.96	−0.068	0.64

* Significant at P < 0.05 (Spearman’s rank correlation was used to find associations of sperm parameters with CAG repeat polymorphism.
